# Onychomadesis and Beau's Line Following Hand-Foot-and-Mouth Disease in a Seven-Year-Old Male

**DOI:** 10.7759/cureus.23832

**Published:** 2022-04-04

**Authors:** Ali Alghamdi, Nadia Mazraani, Yara Alghamdi, Sarah M Albugami

**Affiliations:** 1 Internal Medicine, King Saud Bin Abdulaziz University for Health Sciences, College of Medicine - Western Region, Ministry of National Guard Health Affairs. King Abdullah International Medical Research Center, Jeddah, SAU; 2 Family Medicine, King Abdulaziz Medical City, Ministry of National Guard Health Affairs, Jeddah, SAU; 3 College of Medicine, King Saud Bin Abdulaziz University for Health Sciences, King Abdullah International Medical Research Center, Jeddah, SAU; 4 College of Medicine, King Saud Bin Abdulaziz University for Health Sciences, Jeddah, SAU

**Keywords:** beau's line, coxsackievirus, complication, onychomadesis, hand-foot-and-mouth disease

## Abstract

Hand-foot-and-mouth disease (HFMD) is a viral infection frequently encountered in the pediatric age group. Common culprits in such manifestations are coxsackievirus A16 and human enterovirus 71. The patient presents febrile with erythematous papulovesicular exanthems in the mouth, palms, and soles. HFMD is self-limiting in nature with a rare-complication rate. Onychomadesis is proximal nail separation while Beau’s lines are whitish transverse lines and considered a rare complication of HFMD. Both allude to halted nail-matrix proliferation, and the pathophysiology behind such manifestations is still not yet understood. It is hypothesized that the virus elicits an inflammatory process, inhibiting nail-matrix proliferation or immune-complexes depositing on nails creating an embolism. Onychomadesis and Beau’s lines appear after four to eight weeks of HFMD disease resolution and persist for approximately 35 days. There are no serious sequelae of those manifestations, as the nail basement is still intact. We present a case of a seven-year-old Saudi male presenting with nail changes, mainly onychomadesis and Beau’s lines, after 35 days of HFMD disease resolution. All causes of nail changes have been ruled out and diagnosis of onychomycosis and Beau’s lines secondary to HFDM has been established.

## Introduction

Hand-foot-mouth disease (HFMD) is a relatively common contagious disease in the pediatric age group and is caused by the picornavirus family (coxsackieviruses, mainly coxsackievirus A16 and human enterovirus 71). Patients with HFMD commonly present with fever and erythematous papulovesicular lesions over the palms, soles, and mouth [1]. A study published in Malaysia reported that the incidence of HFMD accounted for 94.3 per 100,000 people in 2017-2018 [2]. The standard treatment is symptomatic due to the low rate of sequelae incidence [1]. Complications are rare; however, some literature report pneumonia, rhabdomyolysis, meningitis, and onychomadesis as rare complications [3]. Onychomadesis is characterized by complete or partial shedding of the fingernails or toenails plate from the matrix with attachment to the nail bed [4]. It is considered a rare complication of HFMD and can be recognized after four to eight weeks from disease onset [1]. Although onychomadesis is mainly referred to as idiopathic, it is associated with a nail-matrix arrest that happens because of medication exposure, autoimmune diseases, neonatal diseases, and infections such as HFMD [4-5]. No invasive or specific intervention is indicated in onychomadesis, and it resolves spontaneously within four to eight weeks [1].

## Case presentation

**Figure 1:**
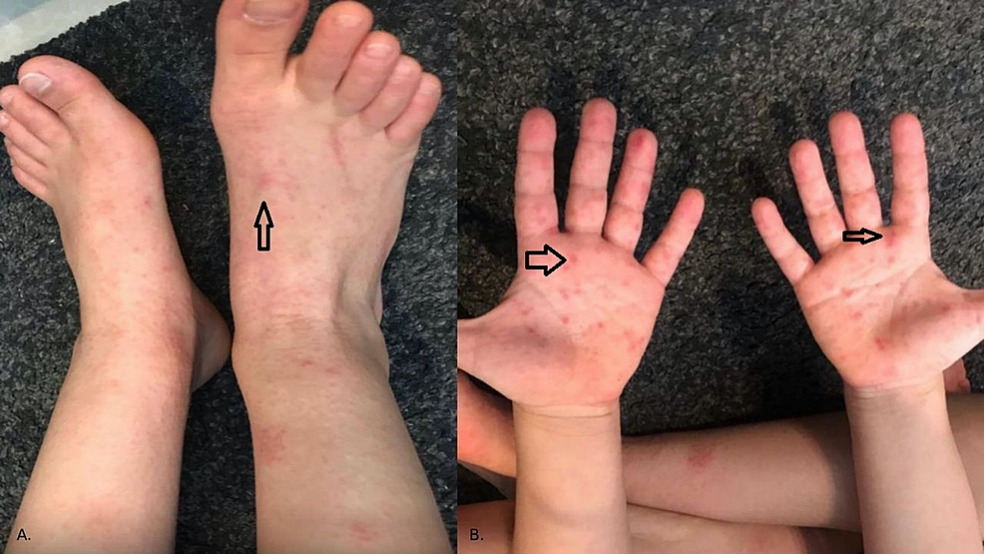
(A) A cluster of erythematous papulovesicular eruptions over the feet; (B) Erythematous papulovesicular eruptions on the palmar surface of both hands

**Figure 2:**
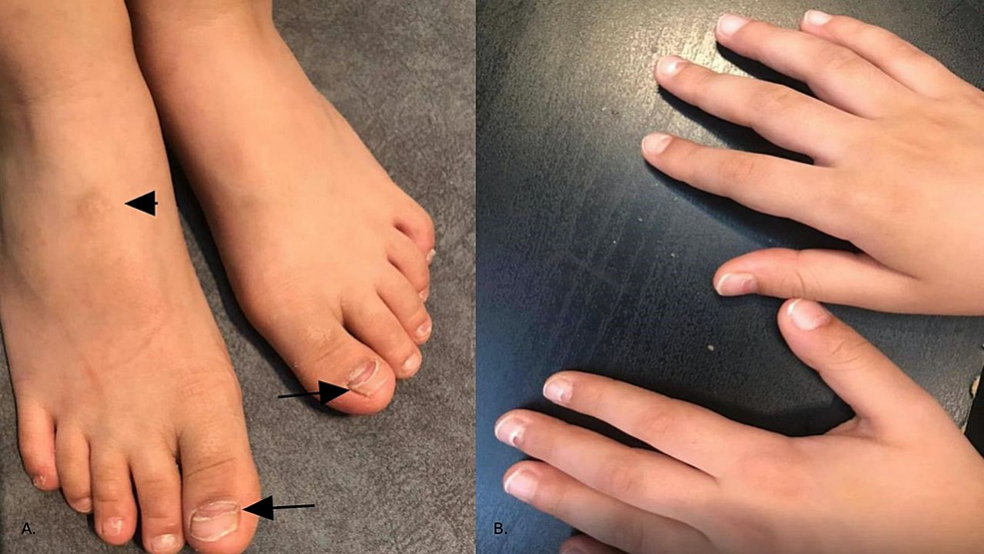
(A) Arrows point to Beau’s lines over the big toes and onychomadesis over the toenails of both feet; arrowheads demarcate post-inflammatory hyperpigmentation following the resolution of HFMD papulovesicular eruptions; (B) On the thumb, index, and middle finger of both hands, there are nail changes, onychomadesis, and Beau’s lines secondary to HFMD HFMD: hand-foot-and-mouth disease

A seven-year-old Saudi male, with no prior significant medical or surgical history, presented to the National Guard Hospital (NGH), in Jeddah, Saudi Arabia, on 28-04-2021, complaining of an itchy rash involving the hand, feet, and buttocks (Figure 1). The rash was accompanied by a sore throat, cough, and fever that lasted a few days. Fever measured at home reaching 41 degrees Celsius. A positive family history of similar complaints was reported from the younger sister who developed fever recently and the youngest brother who had developed a similar rash. Upon examination, the child was found to be active with normal vital signs, and skin examination revealed a papulovesicular erythematous eruption on the palmar and dorsal surfaces of the hand, feet, and buttocks. No obvious vesicles or exudates were observed. Chest examination revealed clear lung sounds and no abnormalities were detected. After proper clinical evaluation, the patient was diagnosed with HFMD; thus, the treatment was supportive only. Five weeks later, on 02-06-2021, the patient presented to the hospital with peeling of skin and loosening of the nails in the fingers and toes (Figure 2). There were no active infections or new medication intake. After clinical correlation with the previous HFMD infection, a diagnosis of onychomadesis and Beau’s lines secondary to HFMD has been established. After researching the subject, it was found to be a rare complication of HFMD. The patient had gained full recovery of the hand and toenails after 10 weeks of patient presentation.

## Discussion

HFMD is a self-limiting, viral infection commonly affecting children less than 10 years; however, adult-onset cases are also observed [5-6]. Patients commonly present with flu-like symptoms like fever, lymphadenopathy, and malaise. Moreover, cutaneous manifestations like erythematous papulovesicular eruptions on the palms, soles, and buttocks, along with ulceration on the mucosal membranes can be seen. The mode of transmission is the fecal-oral route. Coxsackievirus A16 and enteroviruses are commonly implicated in the majority of HFMD cases. Although it is a self-limiting disease and management is symptomatic, late-onset complications have been reported but with no serious sequelae. Those late complications could affect the nail matrix leading to onychomadesis and Beau’s line. Onychomadesis is a proximal separation of the nail bed while Beau’s line is a white transverse line alluding to the halted growth of the nail plate [7-8]. Onychomadesis could be attributed to many factors like trauma, fever, chemotherapy-induced, and medication ingestion. The presence of onychomadesis indicates a severe nail disorder more than Beau’s line [7-8]. Furthermore, when nail matrix proliferation is interrupted, the nail de-attaches from the nail bed. When a new nail grows without connection to the older nail, this leads to proximal nail separation [8]. Nail changes following HFMD infection are a rare phenomenon; however, they are emerging lately, and onychomadesis is more commonly reported in HFMD patients than in Beau’s line [6-10]. Nail changes commonly manifest 30 to 90 days after an acute viral infection episode. Coxsackievirus A16 has been confirmed to be a culprit in patients with onychomadesis secondary to HFMD; enteroviruses have also been implicated but to a lesser extent [7-8]. Nail changes secondary to HFMD are a peculiar complication. Not all nails are affected, and on average, the shedding of four nails is observed [7]. The pathophysiology is not clearly understood to this day; however, many theories have been proposed. Hardin J et al. and Bettoli et al. proposed that the mechanism of onychomadesis could be a direct result of viral infection eliciting an inflammatory process, leading to arrested nail growth, or indirectly through viral immune complexes and distal embolism deposition on the nail matrix [5,10]. However, Chiu et al. proposed that direct injuries to the nail matrix from HFMD cutaneous vesicular lesions lead to onychomadesis [7].

In this case report and literature review, onychomadesis and Beau’s line appeared after symptoms resolution by approximately 35 days. The patient is medically free and does not suffer from any systemic illness that could cause nail disorders. Also, the patient did not ingest any medication that could explain the manifestation of onychomadesis and Beau’s line on their nails. The patient had a transient mild fever that could not, also, explain the nail disorder. The fact that the patient had an acute viral illness and nail manifestation appeared after approximately 35 days allude to onychomadesis and Beau’s line secondary to HFMD. From a review of literature, there were no cases reported from Saudi Arabia about onychomadesis secondary to HFMD. The only case reported was from Beirut about a nine-month-old male with HFMD disease developing onychomadesis as a late complication [9]. The commonly affected population of nail disorders secondary to HFMD is the pediatric population and manifestation of this phenomenon takes 30 to 90 days after an acute viral illness [4-10]. Although no serious sequelae occur from onychomadesis, as the nail basement remains intact, nail shedding is a concern to parents. The exact pathogenesis and mechanism of onychomadesis and Beau’s line following HFMD are not fully understood, and further research should be done on that matter.

## Conclusions

Hand-foot-mouth disease (HFMD) is a common infectious disease in the pediatric age group caused mainly by coxsackieviruses. Patients usually present with erythematous papulovesicular lesions over palms, soles, and mouth in addition to fever. This case report presents a seven-year-old male with onychomadesis and Beau's line after being diagnosed with hand-foot-mouth disease. The reason behind this complication is still not well-known studied, thus more research on this subject is highly needed and required.
